# Higher dietary phytochemical index is associated with lower risk of epithelial ovarian cancer in Chinese women: a case–control study

**DOI:** 10.3389/fnut.2026.1915090

**Published:** 2026-08-28

**Authors:** Yu’e Yang, Ruiqi Zhang, Mengwei Zhu, Yizheng Zu

**Affiliations:** 1 First Clinical Medical School of Gansu University of Chinese Medicine, Lanzhou, Gansu, China; 2 The First Clinical Medical College of Ningxia Medical University, Yinchuan, Ningxia Hui Autonomous Region, China; 3 Department of Reproductive Medicine, The Second Affiliated Hospital of Fujian Medical University, Quanzhou, Fujian, China; 4 Department of Gynecology, The Second People’s Hospital of Changzhou, The Third Affiliated Hospital of Nanjing Medical University, Changzhou, Jiangsu, China

**Keywords:** antioxidants, case–control studies, diet, ovarian neoplasms, phytochemicals

## Abstract

**Background:**

Dietary phytochemicals have been suggested to play a protective role against cancer development. This study aimed to examine the association between dietary phytochemical index (DPI) and epithelial ovarian cancer (EOC) risk among Chinese women.

**Methods:**

In this retrospective case–control study, 500 women (250 newly diagnosed EOC patients and 250 cancer-free participants) were included. Dietary intake was assessed using a validated food frequency questionnaire (FFQ), and DPI was calculated as the percentage of energy derived from phytochemical-rich foods. Multivariable logistic regression models were used to estimate odds ratios (ORs) and 95% confidence intervals (CIs) for the association between DPI and EOC risk.

**Results:**

Higher DPI was significantly associated with lower odds of EOC. Each one-unit increase in DPI was linked to a reduced risk in both crude and adjusted models (fully adjusted OR: 0.90; 95% CI: 0.81–0.99). Participants in the highest DPI tertile had a 31% lower odds of EOC compared to those in the lowest tertile (OR: 0.69; 95% CI: 0.49–0.96). The inverse association was more pronounced among overweight/obese women (*P* for interaction = 0.041).

**Conclusion:**

Greater adherence to a phytochemical-rich diet may be associated with reduced risk of EOC. Prospective studies are warranted to confirm these findings.

## Introduction

Epithelial ovarian cancer (EOC) is a leading cause of gynecologic cancer mortality worldwide, largely because it is often diagnosed at an advanced stage. In China, the age-standardized incidence of ovarian cancer is relatively low (about 3.8 per 100,000) (1), yet the 5-year survival is only approximately 45% due to late detection (2). Established risk factors (e.g., nulliparity, late menopause, family history) explain some cases (3), but modifiable factors remain poorly understood. For example, greater consumption of vegetables and fruits has been associated with substantially lower ovarian cancer risk in some populations (4). Likewise, a U. S. study found that high intake of fiber, total carotenoids, lignans and vegetables was associated with a risk reduction (5).

Phytochemicals, bioactive compounds in plant foods (flavonoids, carotenoids, polyphenols, etc.), are hypothesized to have anticancer effects via antioxidant, anti-inflammatory, and hormone-modulating mechanisms (6). To capture overall dietary exposure to these compounds, McCarty proposed a dietary phytochemical index (DPI), defined as the percentage of daily calories derived from phytochemical-rich foods (fruits, vegetables, whole grains, legumes, nuts/seeds, etc.) (7). In recent years, higher DPI scores have been linked to lower risk of various cancers. A recent meta-analysis of observational studies reported that diets with high DPI were strongly associated with reduced cancer risk overall (8). Likewise, broad plant-based dietary patterns have generally been protective against ovarian cancer. For example, higher scores on plant-based diet indices (favoring healthy plant foods) were associated with a lower ovarian cancer risk (9). Conversely, diets high in pro-inflammatory foods (a low-DPI equivalent) have been linked to higher ovarian cancer odds (10).

Despite this suggestive evidence, the role of dietary phytochemical load, as quantified by DPI, in ovarian cancer risk has not been directly studied in Asian populations. No prior epidemiologic studies have examined DPI in relation to EOC, and few have focused on Chinese women, whose dietary patterns and cancer incidence differ from Western cohorts. Compared with Western dietary patterns, traditional Chinese diets are characterized by greater consumption of soy products, legumes, vegetables, and tea, relatively lower intake of dairy products, and a higher contribution of refined grains, particularly white rice, to total energy intake (11). Furthermore, the epidemiological profile of ovarian cancer differs across populations, with variations in incidence rates, reproductive patterns, lifestyle factors, and environmental exposures between Asian and Western countries (12). Therefore, findings derived primarily from Western cohorts may not be directly generalizable to Chinese populations. Investigating DPI in Chinese women provides an opportunity to evaluate whether a phytochemical-rich dietary pattern is associated with EOC risk within a distinct nutritional and cultural context, thereby extending current evidence beyond Western-based populations. Importantly, most existing studies of diet and ovarian cancer are limited to single nutrients or small case series. A dietary pattern approach, such as DPI, may better capture the cumulative and synergistic effects of multiple phytochemicals and plant-derived foods that may collectively influence carcinogenesis through antioxidant, anti-inflammatory, hormonal, and metabolic pathways. To address this gap, we conducted a retrospective case–control study of DPI and EOC risk in Chinese women.

## Methods

### Study design and population

This retrospective case–control study was conducted at the General Hospital of Ningxia Medical University between March 2021 and March 2026. Ethical approval was approved by Ethics Review Committee of Ningxia Medical University General Hospital (Approval No. KYLL-2021-537). The study adhered to the Declaration of Helsinki, and all participants gave written informed consent before taking part. The study protocol adhered to the guidelines of the Strengthening the Reporting of Observational Studies in Epidemiology (STROBE) statement.

The study population included patients with EOC and cancer-free controls. EOC cases were women aged ≥18 years with a new, histologically confirmed diagnosis of primary epithelial ovarian cancer and had not received any prior treatment, including surgery, chemotherapy, or radiotherapy, before enrollment. Diagnosis was established through a standardized clinical pathway, including gynecological examination, imaging assessment (ultrasound and/or CT/MRI when indicated), and serum tumor marker evaluation (including CA-125), with definitive confirmation based on postoperative histopathological examination of surgical specimens. Tumors were classified according to World Health Organization (WHO) criteria (13), and disease staging was performed using the International Federation of Gynecology and Obstetrics (FIGO) system (14).

Controls were selected from women attending routine health examinations during the same period and were confirmed to be free of any malignancy based on medical history, clinical evaluation, and available medical records. Controls were frequency-matched to cases based on age (±5 years), a commonly adopted approach in epidemiological case–control studies of ovarian cancer (15), and by recruitment period to ensure comparable distributions of key demographic variables. All participants were required to have resided in the study area for at least 1 year, to be capable of completing the study questionnaire, and to provide written informed consent prior to enrollment. Participants with severe systemic conditions such as advanced hepatic, renal, or cardiovascular disease, pregnant or lactating women, and individuals with incomplete, inconsistent, or implausible dietary data, including implausible total energy intake, from the food frequency questionnaire (FFQ), as well as those who declined or were unable to provide written informed consent, were excluded from the final analysis.

### Sample size calculation

The required sample size was calculated using power analysis to detect a significant association between DPI and EOC risk. The required sample size was initially estimated based on available epidemiological evidence regarding dietary indices and cancer risk. Because direct evidence regarding the association between DPI and EOC was unavailable at the time of study design, assumptions were informed by previous nutritional epidemiological research involving antioxidant-related dietary indices and cancer outcomes (16). An odds ratio of 0.60 was considered as a moderate association, with 80% power and a two-sided significance level of 0.05, resulting in an estimated requirement of 248 cases and 248 controls. We acknowledge that effect estimates derived from other cancer types may not fully represent the relationship between dietary patterns and epithelial ovarian cancer; therefore, this assumption should be interpreted as an approximate estimate for study planning. Therefore, a *post hoc* power analysis based on the observed effect size in the present study was also conducted.

### Data collection

Demographic, lifestyle, reproductive, and clinical information were collected using structured questionnaires and medical record reviews. Variables included age, body mass index (BMI), physical activity, smoking status, alcohol consumption, education level, occupation, family history of cancer, parity, menopausal status, oral contraceptive use, hormone replacement therapy, and menstrual characteristics such as ovulatory cycle regularity. Body weight was assessed using a calibrated scale with participants standing upright, barefoot, and wearing light clothing. Height was measured with a stadiometer while participants maintained an erect posture with the head positioned in the Frankfurt plane. BMI was then calculated as weight in kilograms divided by the square of height in meters (kg/m^2^).

### Dietary assessment and calculation of dietary phytochemical index

Dietary intake was assessed using a validated semi-quantitative 149-item Chinese-formatted, culturally adapted FFQ (17), which captured habitual food consumption over a specified reference period prior to diagnosis (for cases) or interview (for controls). Participants reported average frequency and portion sizes of commonly consumed food items. To enhance the precision of dietary intake estimation, the FFQ was complemented with 24-h dietary recall data. Daily nutrient intakes were calculated using information from the 2009 Chinese Food Composition Table (18).

DPI was calculated as the percentage of total daily energy intake derived from foods rich in phytochemicals (7): DPI (%) = (Energy from phytochemical rich foods ∕ Total daily energy intake) × 100. These included fruits, vegetables (except potatoes), whole grains, legumes, nuts, seeds, olive oil, and soy products. Higher DPI values indicated a greater proportion of phytochemical-rich foods in the overall diet, reflecting better diet quality in terms of plant-based food consumption DPI was analyzed both as a continuous variable and in tertiles, with the lowest tertile used as the reference category.

### Statistical analysis

Baseline characteristics were compared between groups using independent sample *t*-tests for continuous variables and chi-square tests for categorical variables. Multivariable logistic regression models were applied to estimate odds ratios (ORs) and 95% confidence intervals (CIs) for the association between DPI and EOC risk. Three models were constructed: Model 1 was unadjusted; Model 2 adjusted for age and total energy intake; and Model 3 further adjusted for potential confounders, including BMI, education, occupation, smoking, alcohol consumption, physical activity, parity, menopausal status, family history of cancer, hormone therapy use, oral contraceptive use, and ovulatory cycle status. Subgroup analyses were conducted to assess potential effect modification by age, BMI, menopausal status, parity, smoking status, physical activity, education level, and family history of cancer. Interaction terms were included in regression models to test for statistical heterogeneity across subgroups. A two-sided *p* < 0.05 was considered statistically significant. All analyses were performed using SPSS software, version 25.0 (IBM Corp., Armonk, NY, United States).

## Results

### Baseline characteristics

A total of 500 women with mean age of 53.1 years were included in the present study, comprising 250 patients with EOC and 250 controls. Baseline characteristics of study participants are presented in Table 1. Compared to the Non-EOC group, the EOC group showed significantly lower physical activity levels (16.5 vs. 18.2 MET-hours/week, *p* = 0.010), were more likely to be overweight or obese (48.0% vs. 38.0%, *p* = 0.012), and had a lower prevalence of irregular ovulatory cycles (4.8% vs. 8.8%, *p* = 0.038).

**Table 1:** Baseline characteristics of participants.

Characteristics	EOC cases (*n* = 250)	Controls (*n* = 250)	*p*-value
Age (years)	53.4 ± 8.6	52.8 ± 8.9	0.412
Energy intake (kcal/day)	1712.5 ± 392.4	1689.3 ± 401.7	0.448
DPI	32 ± 6.77	34.91 ± 6.10	<0.001
Physical activity (MET-hours/week)	16.5	18.2	0.01
BMI (kg/m^2^)			0.012
Normal (<25)	130 (52.0)	155 (62.0)	
Overweight/Obese (≥25.0)	120 (48.0)	95 (38.0)	
Postmenopausal			0.318
No	118 (47.2)	129 (51.6)	
Yes	132 (52.8)	121 (48.4)	
Parity			0.489
No (nulliparous)	12 (4.8)	9 (3.6)	
Yes (≥1 child)	238 (95.2)	241 (96.4)	
Oral contraceptive use			0.823
No	198 (79.2)	200 (80.0)	
Yes	52 (20.8)	50 (20.0)	
Irregular ovulatory cycles			0.038
No	238 (95.2)	228 (91.2)	
Yes	12 (4.8)	22 (8.8)	
Hormone replacement therapy			0.735
No	245 (98.0)	246 (98.4)	
Yes	5 (2.0)	4 (1.6)	
Education ≥ college			0.591
No	218 (87.2)	214 (85.6)	
Yes	32 (12.8)	36 (14.4)	
Occupation			0.856
No	122 (48.8)	120 (48.0)	
Yes	128 (51.2)	130 (52.0)	
Smoking status			0.213
Never smoker	210 (84.0)	220 (88.0)	
Ever smoker	40 (16.0)	30 (12.0)	
Alcohol consumption			0.607
No	241 (96.4)	243 (97.2)	
Yes	9 (3.6)	7 (2.8)	
Family history of any cancer			0.196
No	172 (68.8)	185 (74.0)	
Yes	78 (31.2)	65 (26.0)	
Family history of breast/ovarian cancer			0.761
No	244 (97.6)	245 (98.0)	
Yes	6 (2.4)	5 (2.0)	
DPI tertile			0.021
T1	105 (42.0)	83 (33.2)	
T2	85 (34.0)	83 (33.2)	
T3	60 (24.0)	84 (33.6)	

Data were expressed as mean ± SD and percent (%) for continuous and categorical variables, respectively. *p*-values were calculated based on independent sample *t*-test or Chi-square test. Abbreviation: DPI, Dietary Phytochemical Index; EOC, Epithelial Ovarian Cancer; BMI, Body Mass Index; MET, metabolic equivalent tasks.

### Association between DPI and odds of EOC

Multivariable logistic regression analyses evaluating the association between DPI and EOC risk are shown in Table 2. In continuous analyses, each one-unit increase in DPI was associated with a 12% lower odds of EOC in the crude model (OR: 0.88; 95% CI: 0.79–0.98; *p* = 0.021). This inverse association remained statistically significant after adjustment for age and energy intake (OR: 0.89; 95% CI: 0.80–0.98; *p* = 0.028), and after further adjustment for all potential covariates (OR: 0.90; 95% CI: 0.81–0.99; *p* = 0.047). Additionally, when DPI was analyzed by tertiles, participants in the highest tertile (T3) had significantly lower odds of EOC compared with those in the lowest tertile (T1) in the fully adjusted model (OR: 0.69; 95% CI: 0.49–0.96; *p* = 0.034).

**Table 2:** The association between dietary phytochemical index (DPI) and epithelial ovarian cancer among Chinese women.

Characteristic	Model 1*	*p*-value	Model 2**	*P*-value	Model 3***	*P*-value
Continuous DPI	0.88 (0.79–0.98)	0.021	0.89 (0.80–0.98)	0.028	0.90 (0.81–0.99)	0.047
DPI tertiles						
T1	Reference		Reference		Reference	
T2	0.78 (0.52–1.16)	0.218	0.80 (0.53–1.20)	0.273	0.82 (0.54–1.25)	0.351
T3	0.58 (0.38–0.86)	0.010	0.63 (0.42–0.91)	0.019	0.69 (0.49–0.96)	0.034
P for trend	0.009		0.016		0.031	

*Model 1 was unadjusted, **Model 2 was adjusted for age and energy intake, and ***Model 3 was adjusted for: age, energy intake, education status, occupational status, smoking status, alcohol consumption and body mass index, physical activity, parity, menopausal status, family history of cancer, hormone therapy status, ovulatory cycles status and oral contraceptive use. DPI, Dietary Phytochemical Index; OR, Odds Ratio; CI, Confidence Interval.

### Subgroup analyses

Subgroup analyses are presented in Table 3. The inverse association between DPI and EOC risk was generally more pronounced among older, postmenopausal, overweight/obese, parous, never-smoking, and physically inactive women. BMI was the only significant effect modifier of this association (*P* for interaction = 0.041), indicating a stronger inverse association among overweight/obese participants.

**Table 3:** Associations between dietary phytochemical index (DPI) and epithelial ovarian cancer, stratified by selected factors.

Subgroups	OR (95% CI)	*P*-value	P for interaction
Age			0.287
<55 years	0.68 (0.39–1.17)	0.163	
≥55 years	0.54 (0.30–0.97)	0.039	
BMI (kg/m^2^)			0.041
Normal (<25.0)	0.72 (0.42–1.23)	0.228	
Overweight/Obese (≥25.0)	0.48 (0.26–0.87)	0.015	
Menopausal status			0.118
Premenopausal	0.70 (0.38–1.29)	0.251	
Postmenopausal	0.55 (0.32–0.95)	0.032	
Parity			0.364
Nulliparous/0 child	0.79 (0.21–2.94)	0.728	
≥1 child	0.60 (0.38–0.95)	0.029	
Smoking status			0.209
Never smoker	0.63 (0.40–0.99)	0.045	
Ever smoker	0.52 (0.21–1.29)	0.157	
Physical activity			0.173
Low	0.50 (0.28–0.91)	0.023	
Moderate/high	0.70 (0.41–1.18)	0.182	
Family history of cancer			0.326
No	0.65 (0.39–1.08)	0.098	
Yes	0.51 (0.25–1.04)	0.064	
Oral contraceptive use			0.402
No	0.64 (0.40–1.02)	0.061	
Yes	0.49 (0.19–1.28)	0.146	
Education level			0.447
<College	0.63 (0.39–1.01)	0.055	
≥College	0.53 (0.21–1.31)	0.167	

Each stratified model was adjusted for age, sex, BMI, menopausal status, parity, smoking status, physical activity, family history of cancer, oral contraceptive use and education level, except for the stratification variable itself. BMI, body mass index; OR, odds ratio; CI, confidence interval.

### Power analysis

Assuming a prevalence of high DPI exposure of approximately 33% among controls, the corresponding exposure prevalence among cases was estimated at 22.2%. With 250 cases and 250 controls and a two-sided *α* level of 0.05, the achieved statistical power was approximately 79%, suggesting that the study had adequate power to detect the observed association.

## Discussion

In this retrospective case–control study, we observed that a higher DPI, reflecting a diet rich in fruits, vegetables, whole grains, legumes, nuts and other plant-derived foods, was significantly associated with lower risk of EOC. In fully-adjusted models, women in the highest DPI tertile had an approximately 30% lower odds of EOC compared to those in the lowest tertile, with a clear trend across tertiles. This inverse association persisted after controlling for demographic, reproductive and lifestyle factors. Although the magnitude of this association should be interpreted cautiously due to the observational design, the consistency of the findings across continuous and categorical analyses suggests that overall dietary quality, rather than individual nutrients alone, may play a relevant role in ovarian cancer prevention. Given the multifactorial nature of ovarian carcinogenesis, dietary patterns incorporating multiple bioactive compounds may exert cumulative protective effects that cannot be fully captured by examining single foods or nutrients.

The finding aligns with the limited literature on phytochemical-rich diets and ovarian cancer. In a recent meta-analysis of observational studies, Ahmadirad et al. reported that the highest DPI was associated with substantially reduced risk of overall cancer (8). Although that analysis included diverse cancers, it underscores a consistent inverse link between phytochemical intake and malignancy risk. More specific to ovarian cancer, McCann et al. found that women in the highest quintile of carotenoids, dietary fiber or lignans had markedly lower EOC risk (5). Similarly, Zhang et al. reported a strong protective effect of high vegetable and fruit intake against EOC, whereas high animal fat and salted vegetable intake greatly increased risk (4). Together, these results, including our own, suggest that diets rich in plant-based, phytochemical-containing foods are linked to lower ovarian cancer incidence, consistent with general dietary recommendations for cancer prevention.

From a public health perspective, these findings support the promotion of dietary patterns emphasizing diverse plant-based foods as a potentially modifiable strategy for reducing ovarian cancer risk. Importantly, such dietary recommendations may provide additional benefits beyond cancer prevention, including improvements in metabolic health, weight management, cardiovascular risk reduction, and inflammatory status. Therefore, encouraging adherence to phytochemical-rich dietary patterns may represent a feasible component of integrated cancer prevention strategies, particularly among populations with increasing burdens of obesity and metabolic disorders. However, given the observational design of this study, a causal relationship cannot be established, and the findings should therefore be interpreted with caution.

Our findings also complement recent work on holistic plant-based diet patterns. For example, a case–control study in Italy evaluated “healthy” versus “unhealthy” plant-based diet indices and found that high adherence to a healthy plant-based diet (characterized by abundant vegetables, fruits, whole grains, legumes and nuts) was associated with reduced ovarian cancer risk, whereas an “unhealthy” plant diet was associated with higher risk (9). These pattern-based studies reinforce the idea that not just any plant foods, but quality plant foods (rich in phytochemicals and fiber) are beneficial. In our study, the DPI serves as an index of overall phytochemical load. The inverse DPI–EOC association we observed is thus in line with accumulating evidence that plant-based diets confer protection in hormone-related cancers, likely through multiple synergistic mechanisms (19, 20).

There are plausible pathways by which a phytochemical-rich diet could lower ovarian cancer risk. Whole plant foods contain numerous bioactive compounds (flavonoids, carotenoids, phenolic acids, phytoestrogens, etc.) that exert antioxidant, anti-inflammatory and hormonal effects. Phytochemicals can neutralize reactive oxygen species and upregulate endogenous antioxidant defenses, thereby reducing DNA oxidative damage (21). For instance, dietary carotenoids, polyphenols and isoflavones have all been shown to inhibit tumorigenesis in experimental models (22–24). Plant foods also induce phase II detoxifying enzymes and inhibit certain phase I enzymes (25), which can enhance clearance of carcinogens. Together, these antioxidant and chemoprotective actions can slow mutation accumulation and malignant transformation. Phytochemicals may further act via hormonal and metabolic modulation. Fiber-rich plant diets alter estrogen metabolism and insulin dynamics. Fermentation of fiber by gut microbes produces short-chain fatty acids that improve insulin sensitivity and attenuate systemic inflammation (26). A healthy microbiome also regulates estrogen levels. Gut bacterial β-glucuronidase can deconjugate estrogens, affecting enterohepatic recycling and circulating estrogen availability (27). By contrast, diets high in animal fat and low in fiber can prolong estrogen exposure and increase insulin-like growth factor signaling, both of which promote ovarian carcinogenesis (28).

We observed stronger DPI–EOC associations in certain subgroups (older women, postmenopausal, overweight/obese, parous, never-smokers, and sedentary). Notably, BMI was a significant effect modifier, so that the inverse association was most pronounced among overweight/obese women. One possible interpretation is that overweight individuals have higher baseline inflammation and circulating estrogens (due to adipose aromatization) (29). Similarly, postmenopausal women depend more on peripheral estrogen conversion (which can be modulated by diet and gut flora) and less on ovarian hormones (30). While these interactions suggest differential susceptibility, they could also reflect residual confounding (e.g., smokers may have generally poorer diet) or chance findings, given multiple subgroup tests.

### Strengths, limitation, and future directions

As key strengths, all ovarian cancer cases were histologically confirmed, ensuring diagnostic accuracy. We used a validated FFQ, supplemented by 24-h recalls, to capture habitual diet; this approach tends to reduce random measurement error relative to FFQ alone. Importantly, we adjusted for a comprehensive set of confounders (age, energy intake, BMI, reproductive factors, family history, oral contraceptive use, etc.), reducing the chance that the DPI–EOC link is spurious. Our findings are also supported by sensitivity analyses and a clear dose–response trend, strengthening causal inference.

Nevertheless, several limitations merit discussion. By design, our study is retrospective and observational, so we cannot establish causality or temporality; dietary habits were assessed after diagnosis (for cases), and recall bias is possible. Cases may have modified or misremembered their diet in the lead-up to diagnosis, and although we excluded proxy respondents, differential reporting remains a concern. However, since cases were newly diagnosed and interviewed soon after diagnosis, major diet changes are less likely to account for the findings. Selection bias is another issue. Our controls were hospital-based (albeit for non-neoplastic conditions) rather than population controls, which may limit generalizability and could bias results if control diets differ systematically. We attempted to minimize this by excluding controls with long-term dietary modifications and matching on age and center. Measurement error in DPI is also possible; the phytochemical content of foods was not measured directly, and the index assumes that plant-based calorie proportion reflects phytochemical intake. Furthermore, an important limitation of DPI is that it primarily captures the quantity of energy derived from phytochemical-rich foods rather than the overall quality of plant-based dietary patterns. Therefore, DPI may not fully distinguish between high-quality plant foods (such as whole grains, fresh fruits, vegetables, legumes, and nuts) and potentially less beneficial plant-derived foods (such as refined grains, fruit juices, or processed plant foods). Consequently, the protective association observed with higher DPI may partly reflect differences in overall dietary quality rather than phytochemical exposure alone. Future studies incorporating complementary dietary indices, such as healthy and unhealthy plant-based diet indices (hPDI and uPDI), may provide a more comprehensive understanding of how plant food quality influences ovarian cancer risk. Unmeasured or residual confounders (e.g., detailed socioeconomic status, environmental exposures, or genetic predisposition) could also influence the association. Finally, the findings are specific to Chinese women and dietary patterns; applicability to other populations (with different diets or genetics) may be limited. Despite these caveats, the internal consistency of our results and their agreement with external evidence lend credibility to the observed inverse relationship.

Future prospective cohort studies with repeated dietary assessments before cancer diagnosis are warranted to confirm the temporal relationship between DPI and ovarian cancer development and to minimize recall bias. In addition, mechanistic studies integrating biomarkers of inflammation, oxidative stress, estrogen metabolism, gut microbiota composition, and phytochemical exposure could further clarify the biological pathways underlying this association. Randomized dietary intervention studies, although challenging for cancer outcomes, may also help determine whether improvements in dietary quality translate into measurable changes in intermediate biomarkers related to ovarian carcinogenesis.

## Conclusion

Higher DPI was associated with lower odds of epithelial ovarian cancer among Chinese women, with a consistent inverse relationship observed after adjustment for potential confounders. The association appeared stronger in certain subgroups, particularly among overweight or obese individuals. These findings suggest that adherence to a phytochemical-rich dietary pattern may represent a promising, modifiable lifestyle approach for ovarian cancer risk reduction. However, given the retrospective observational design, the results should not be interpreted as evidence of causality. Further prospective studies in diverse populations are needed to validate these findings and clarify whether improving dietary quality can contribute to primary prevention strategies for epithelial ovarian cancer.

## Data Availability

The raw data supporting the conclusions of this article will be made available by the authors, without undue reservation.
